# Correlation of alteration of HLA-F expression and clinical characterization in 593 brain glioma samples

**DOI:** 10.1186/s12974-019-1418-3

**Published:** 2019-02-12

**Authors:** Enshan Feng, Tingyu Liang, Xiaoyong Wang, Juan Du, Kai Tang, Xiaoxuan Wang, Fang Wang, Gan You

**Affiliations:** 1 Department of Neurosurgery, Beijing Ditan Hospital, Capital Medical University, Beijing, 100015 China; 2 Institute of Infectious Diseases, Beijing Ditan Hospital, Capital Medical University, Beijing, 100015 China; 3 Department of Neurosurgery, Beijing Tiantan Hospital, Capital Medical University, No. 6 Tiantan Xili, Dongcheng District, Beijing, 100050 China; 4 Capital Medical University, Beijing, 100069 China

**Keywords:** HLA-F, Glioma, Immunotherapy, OS

## Abstract

**Background:**

Human gliomas are highly fatal tumors with a significant feature of immune suppression. The association of the immune system in gliomas is gradually revealed, and immunotherapy is expected to improve the survival of glioma patients. In-depth understanding of the immune microenvironment of gliomas and their associated immunotherapy was increased exponentially in recent years. Gliomas provide clinical targets for immunotherapy during the search of key regulators of immune response. Our study focused on the human leukocyte antigen (HLA) system that is responsible for regulating the immune system, and discovered the relationship between HLA-F expression and clinical prognosis in gliomas.

**Methods:**

A total of 593 patients with gliomas were included in our research. Of these, 325 patients were from the Chinese Glioma Genome Atlas (CGGA) and 268 were from the GSE 16011 set. Kaplan-Meier (KM) analysis was performed to explore the prognostic value of HLA-F. *t* test analysis was used to find the distribution difference in various groups. R language packages are used for other statistical computations and figure drawing.

**Results:**

HLA-F was negatively correlated with overall survival (OS) in all grades of glioma and glioblastoma (GBM). Moreover, HLA-F was enriched in GBM and isocitrate dehydrogenase 1 wild-type (IDH1 wt) group and considered HLA-F as a mesenchymal subtype marker. Pearson correlation test showed that HLA-F was correlated with other HLA-I molecules.

**Conclusion:**

HLA-F expression was positively correlated with malignant phenotype and negatively correlated with OS, indicating that HLA-F could predict the immune state of gliomas and might be a clinical target of glioma immunotherapy.

## Introduction

Glioma is the most common and malignant brain tumor, accounting for 46% of all intracranial tumors and 2% of all adult cancers [1, 2]. Based on the 2007 World Health Organization (WHO) classification of gliomas, these are classified from WHO grade I to WHO grade IV [3]. The invasion of tumor cells is gradually increased with the progression of tumor grade [4]. Despite the advanced therapeutic methods, the survival time of gliomas show no significant improvement from the past four decades; especially for glioblastoma (GBM), the median survival time is nearly 14 months [5, 6]. The unfavorable prognosis is largely attributed to the escape of immune surveillance and suppression of the immune system [7]. Hence, it is necessary to uncover the regulators of the immune system in gliomas to improve the patients’ survival time.

Although the central nervous system (CNS) is originally considered to be an immune-privileged site, more recent studies discover that it is an immunologically specialized site [8, 9]. Immunotherapy by reversing the suppressed immune status in tumors becomes a promising anticancer therapy [10]. Regulators of immune escape are prevented effectively by current immunotherapies to eliminate gliomas [11]. These defects in surveillance of gliomas could be due to the range of alterations of human leukocyte antigen (HLA) class I alleles [12, 13], dysfunction of T cell receptor (TCR) [14, 15], and abnormal expression of co-stimulatory ligands on glioma cells.

HLA-F is located at the terminal end of chromosome 6, is one of the non-classical HLA class Ib molecules, and is considered as a tolerogenic molecule [16]. HLA-F is a ligand for KIR receptors in natural killer cells (NK cells) and CD8^+^ T cells. HLA-F interacts physically and functionally with three KIR receptors: KIR3DL2, KIR2DS4, and KIR3DS1. HLA-F regulates immunity during infection [17], pregnancy [18], and autoimmunity [19] through NK cell receptors.

HLA-F was reported to be involved in the immunomodulatory effect of visceral system tumors. For example, Lin et al. reported that HLA-F expression was negatively associated with the overall survival (OS) in non-small cell lung cancer patients [20]. Ishigami et al. showed that HLA-F expression was upregulated in gastric cancer [21]. But the function of HLA-F in gliomas remains unknown. In our research, we used the Chinese Glioma Genome Atlas (CGGA) RNA-seq set as a training set and validated the results in the GSE 16011 array set. We hypothesiz that HLA-F plays as an oncogene in glioma and is suggested as a new potential biotarget for therapy.

## Methods

### Patients and data collection

HLA-F expression is downloaded from CGGA which includes 109 grade II, 72 grade III, and 144 grade IV and from the GSE 16011 array set which includes 24 grade II, 85 grade III, and 159 grade IV. To further validate the protein expression of HLA-F in different grades, we randomly selected 15 different grade glioma patients (5 patients for each grade) from Beijing Ditan Hospital for immunohistochemistry (IHC). All the patients are histologically diagnosed by two neuropathologists according to the 2007 WHO classification guidelines, and clinical information is downloaded from each website. This research is approved by the Ethics Committee of Beijing Ditan Hospital.

### Detection of biomarkers of gliomas

Isocitrate dehydrogenase 1 (IDH1) mutations are the most common mutations in gliomas, especially in low-grade gliomas and secondary GBM [22]. In the CGGA RNA-seq set, the genomic DNA was isolated from frozen tissues using a QIAamp DNA Mini Kit (Qiagen) according to the manufacturer’s protocol [22]. IDH1 status in the CGGA RNA-seq set was tested by pyrosequencing. TCGA subtype was based on previous research [23].

### Immunohistochemistry for HLA-F expression

IHC was performed to detect HLA-F protein expression. The tumor tissues excised during the operation were immediately placed in 10% formalin for fixation, followed by dehydration, paraffin embedding, and sectioning. Anti-HLA-F antibody (Abcam) was used at a dilution of 1:100. Each slide was individually reviewed and scored by two independent neuropathologists, HLA-F protein expression (semi-quantitative scoring) = expression intensity × expression area. Expression intensity was scored using a 4-point scale from 0 to 3. Expression area was scored using a 5-point scale from 0 to 4.

### Related signature identification

Significantly related genes with HLA-F expression were retrieved by using the Pearson correlation analysis. Gene Ontology (GO) and Kyoto Encyclopedia of Genes and Genomes (KEGG) analyzed the related gene sets and relevant biological function on the DAVID website (http://david.ncifcrf.gov/). In addition, the heatmap package of R language was used to list the genes positively related with HLA-F expression.

### Statistical analysis

We performed Kaplan-Meier analysis to explore the prognostic value of HLA-F. Cox proportional hazards model analysis was performed to verify HLA-F as an independent prognostic factor. OS was estimated from the date of diagnosis to the date of either death or last follow-up. Student’s *t* test was performed to explore the expression distribution in different groups. Pearson correlation analysis was used to find genes related to HLA-F expression. R language packages (ggplot2, pheatmap, pROC, and corrgram) are used for other statistical computations and drawing figures. All differences were considered statistically significant at the level of *p* < 0.05.

## Results

### HLA-F mRNA expression is upregulated in high-grade gliomas and downregulated in IDH1 mutation gliomas

**Fig. 1:**
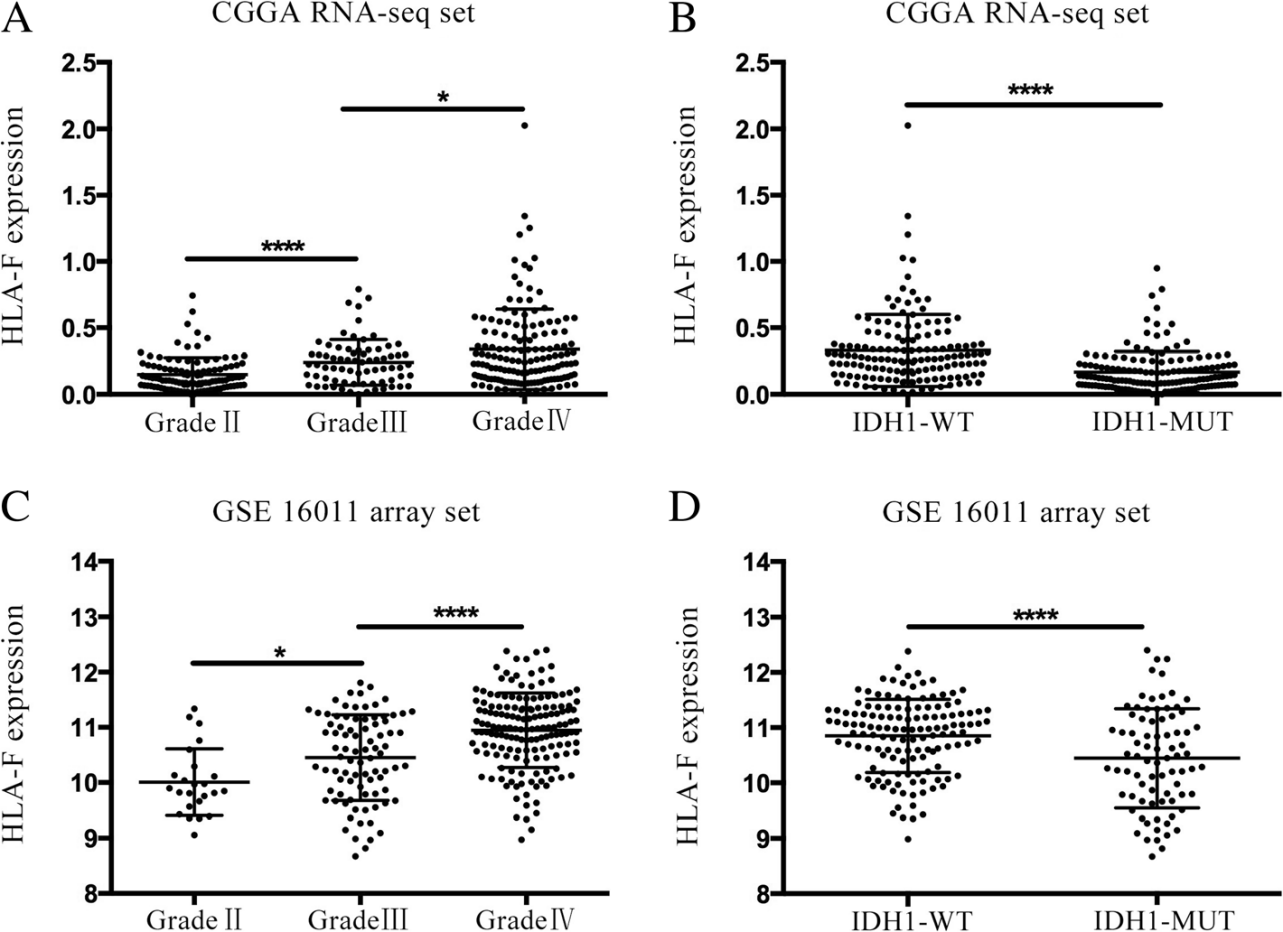
HLA-F mRNA expression pattern in CGGA RNA-seq and GSE 16011 array datasets. HLA-F is enriched in high-grade gliomas in CGGA RNA-seq and GSE 16011 array sets (**a**, **c**). HLA-F is enriched in IDH1 wt gliomas in CGGA RNA-seq and GSE 16011 array sets (**b**, **d**). *p < 0.05 and ****p < 0.0001

**Fig. 2:**
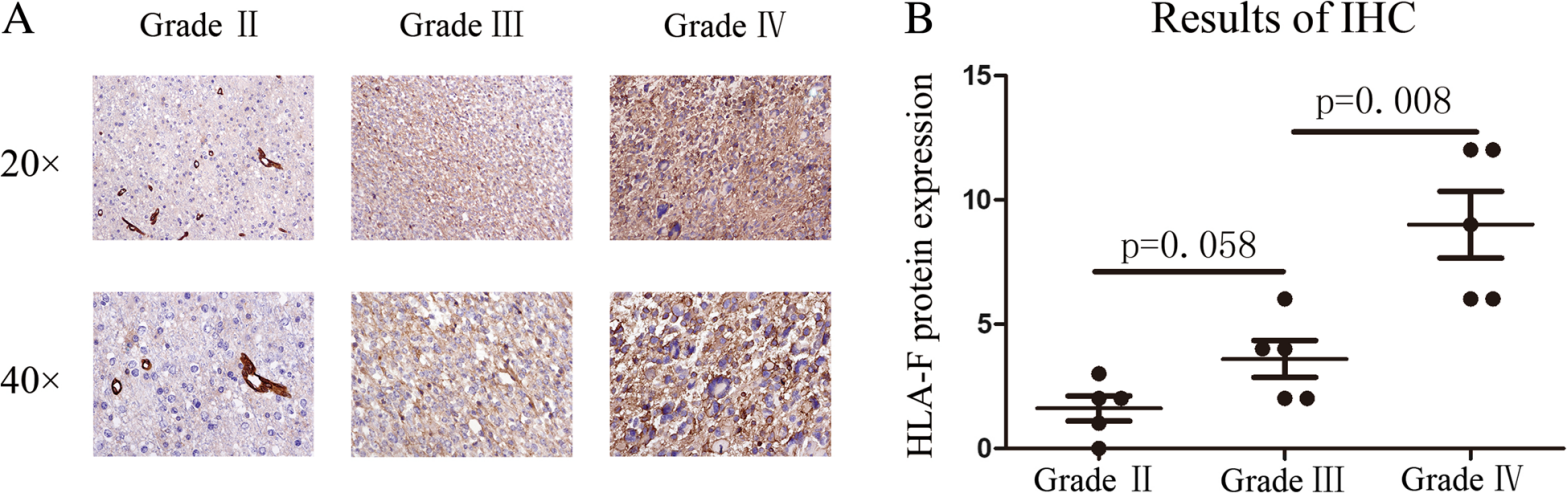
IHC staining of HLA-F in different grades (**a**): upper, × 20; lower, × 40. Analysis of HLA-F protein expression (semi-quantitative scoring, expression intensity × expression area) indicated that HLA-F protein expression is higher in high-grade samples (**b**)

Firstly, 325 samples with HLA-F expression profile and whole follow-up information are obtained from the CGGA RNA-seq set. The GSE 16011 array set was used for validation. In Fig. 1a, the results showed that HLA-F mRNA expression was positively associated with grade (*p* < 0.05). Moreover, HLA-F expression was higher in the isocitrate dehydrogenase 1 wild-type (IDH1 wt) group than in the IDH1 mutation (IDH1 mut) group (*p* < 0.05, Fig. 1b). Fortunately, all the above results were validated in 268 samples from the GSE 16011 array set (Fig. 1c, d). To further explore the relation between tumor grade and HLA-F protein expression, immunohistochemistry (IHC) was performed (Fig. 2a, b), and the results showed that the protein expression of HLA-F (semi-quantitative scoring) was higher in high-grade gliomas (*p* < 0.05).

### HLA-F is a potential marker for mesenchymal molecular subtype gliomas

**Fig. 3:**
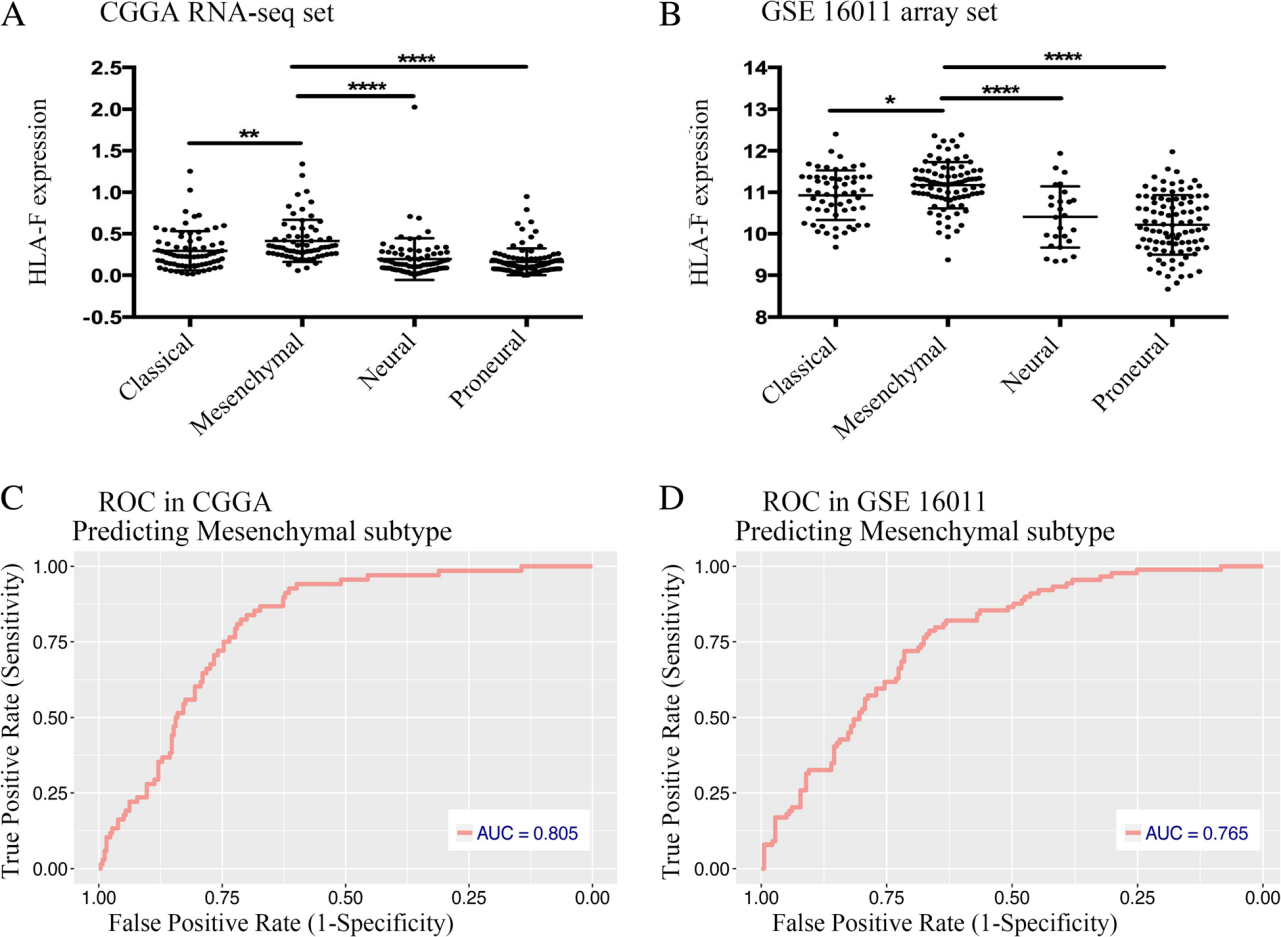
HLA-F expression in different molecular subtypes of the TCGA transcriptional classification scheme in CGGA (**a**) and GSE 16011 datasets (**b**). ROC curves of HLA-F expression to predict mesenchymal subtype in CGGA (**c**) and GSE 16011 (**d**) datasets. *p < 0.05, **p < 0.01, and ****p < 0.0001

To explore the expression distribution of HLA-F, we evaluated the expression pattern of HLA-F in different molecular subtypes as defined by the Cancer Genome Atlas (TCGA) network [23]. The results (Fig. 3a, b) showed that HLA-F was significantly upregulated in the mesenchymal molecular subtype than in other subtypes in both the CGGA RNA-seq set and GSE 16011 array set. To further validate this finding, receiver operating characteristic curves (ROC) for HLA-F expression and mesenchymal subtype of all grade gliomas are performed. Interestingly, the areas under the curves (AUC) are 80.5% and 76.5% in the CGGA RNA-seq set and GSE 16011 array set, respectively (Fig. 3c, d).

### HLA-F is an independent predictive marker of OS in patients with malignant gliomas

**Fig. 4:**
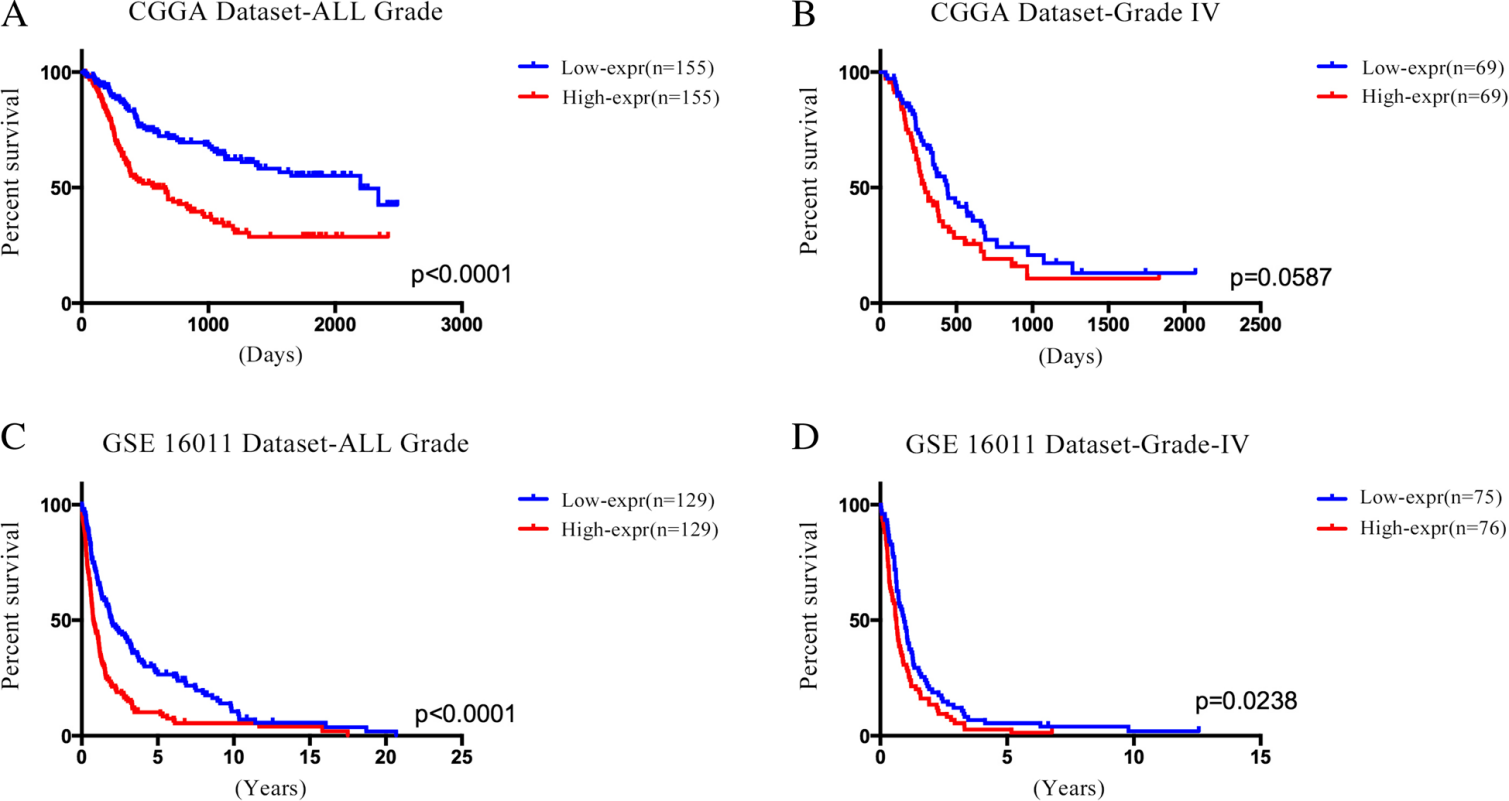
HLA-F mRNA expression was related to clinical outcomes in gliomas. **a**, **b** Kaplan-Meier estimates of survival for all grade and GBM patients in the CGGA RNA-seq set. HLA-F expression was negatively associated with OS of all grade gliomas and GBM (*p* < 0.05). **c**, **d** In the GSE 16011 array set, patients with HLA-F high expression had shorter OS than those with HLA-F low expression (*p* < 0.05)

All 593 patients from the CGGA RNA-seq set and GSE 16011 array set have HLA-F mRNA expression information, and the median value was used as the cutoff point (0.2 for CGGA, 10.8 for GSE 16011). Then, we evaluated the survival time of each group and found that the patients with higher HLA-F expression have remarkably shorter OS in all grade gliomas and GBM in the CGGA RNA-seq set (Fig. 4a, b; *p* < 0.0001 for all grade gliomas, *p* = 0.0587 for GBM). Similar results were obtained from the GSE 16011 array set (Fig. 4c, d; *p* < 0.0001 for all grade gliomas, *p* = 0.0238 for GBM). Then, to verify HLA-F expression as an independent prognostic factor (Table 1), univariate and multivariate Cox regression analyses were performed in CGGA RNA-seq. The results of univariate regression showed that HLA-F (*p* < 0.001) along with grade (*p* < 0.0001), age (*p* < 0.0001), and IDH1 status (*p* < 0.0001) predicted the OS in all grade gliomas. In multivariate regression, HLA-F showed significant results (*p* = 0.027) after adjusting for grade (*p* < 0.0001), age (*p* = 0.687), and IDH1 status (*p* = 0.023). The above results suggested that HLA-F plays an important role in the progression of gliomas. Next, biologic function analysis should be performed to further validate our findings.

**Table 1:** Univariate and multivariate Cox analysis in CGGA RNA-seq set

Clinical factors	Univariate			*p*	Multivariate			*p*
	HR	95%CI			HR	95%CI		
		Lower	Upper			Lower	Upper	
Age	1.039	1.023	1.056	< 0.0001	1.003	0.987	1.020	0.687
Gender (male)	1.204	0.837	1.731	0.316				
IDH1 status (mutation)	0.253	0.172	0.370	< 0.0001	0.588	0.373	0.929	0.023
Grade (grade IV)	3.569	2.751	4.629	< 0.0001	2.923	2.191	3.900	< 0.0001
High HLA-F expression	5.420	3.335	8.807	< 0.0001	2.069	1.087	3.941	0.027

### HLA-F is associated with immune functions in gliomas

**Fig. 5:**
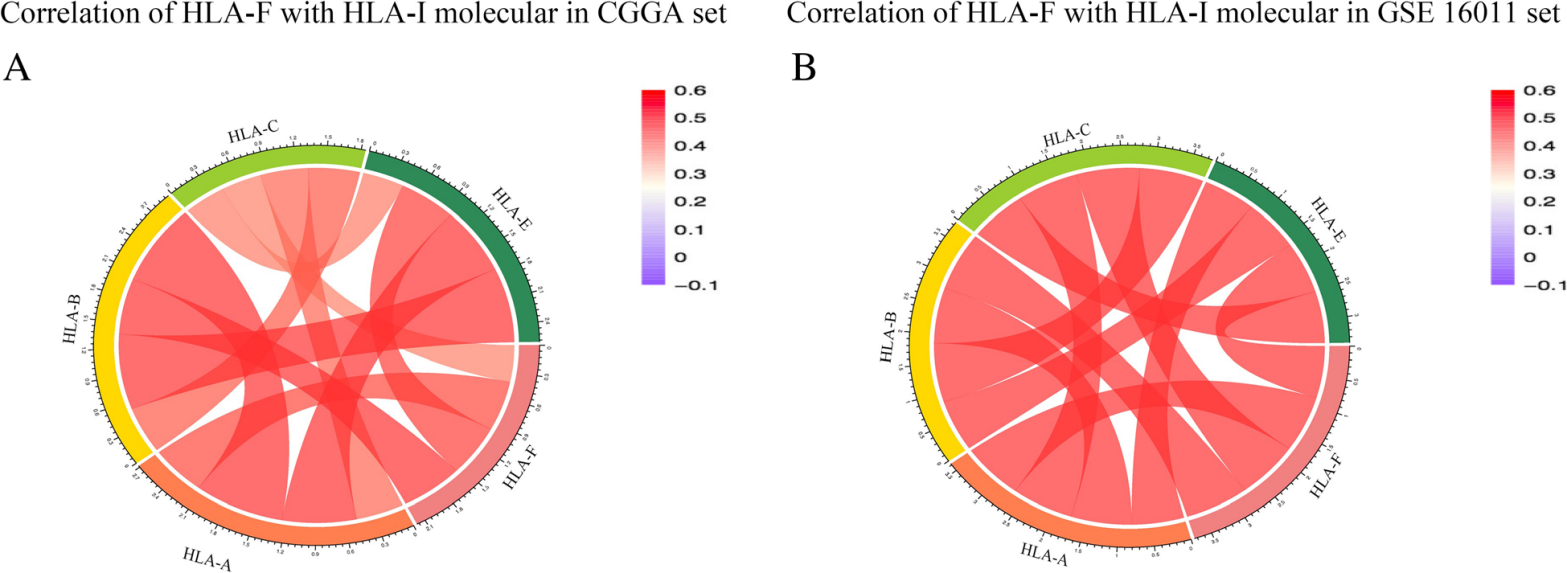
Correlation of HLA-F with HLA-I molecular in the CGGA RNA-seq set (**a**) and GSE 16011 array set (**b**)

**Fig. 6:**
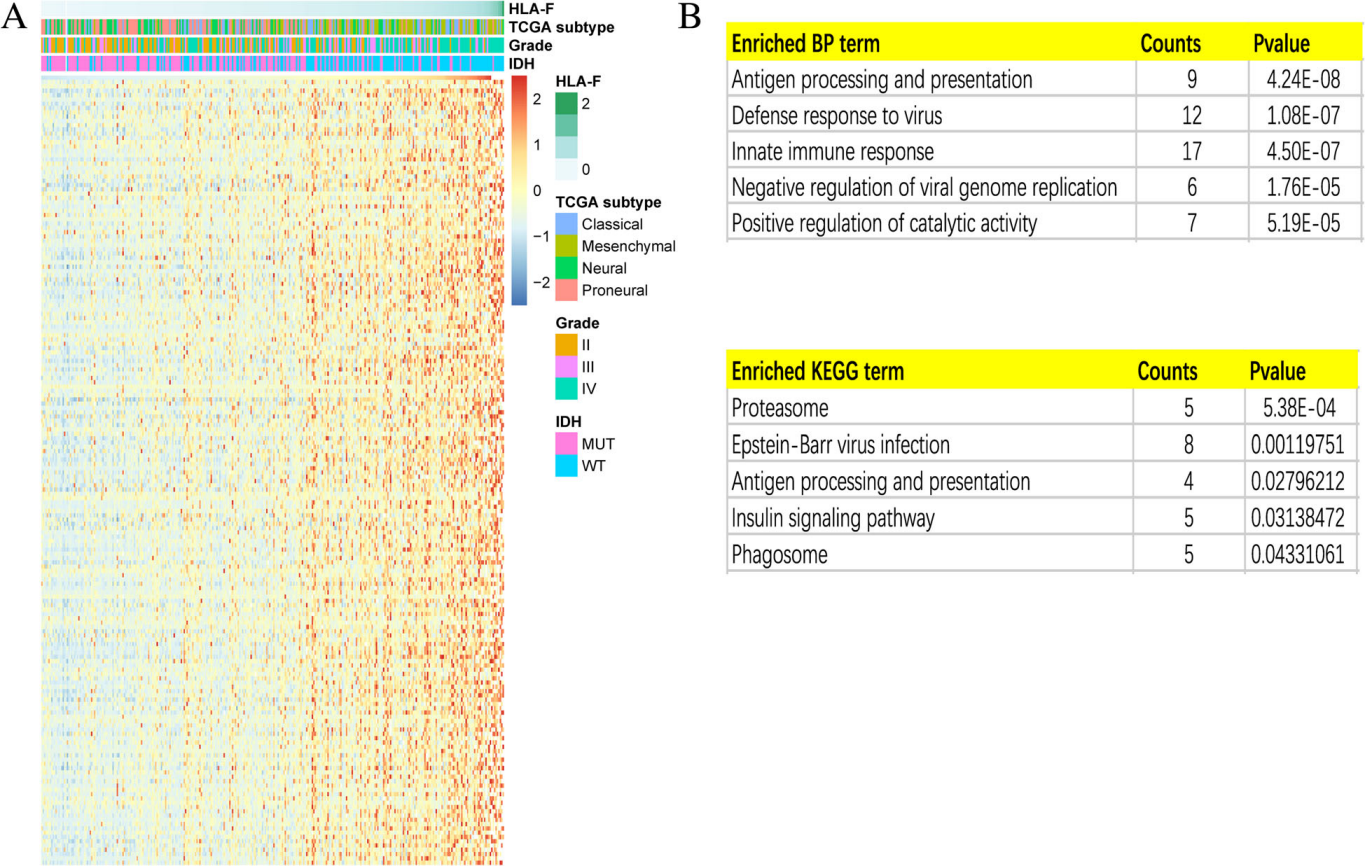
One hundred eighty-three genes positively related (*R* > 0.5) with HLA-F expression (**a**), and the BP and KEGG analysis results show that HLA-F expression is related to immune function of gliomas (**b**)

To investigate the relationship of HLA-F and another HLA-I molecular family, we performed Pearson correlation analysis in the CGGA RNA-seq set and GSE 16011 array set. The results (Fig. 5a, b) showed that the HLA-F expression was significantly associated with HLA-A, HLA-B, HLA-C, and HLA-E. To explore the biological processes associated with HLA-F expression in gliomas, Pearson correlation analysis between HLA-F expression and other genes in whole genome gene profiling of 325 patients in the CGGA RNA-seq set was performed. The results revealed that 183 genes (*R* > 0.5) are positively related to HLA-F expression (Fig. 6a). Biological process (BP) and KEGG analysis are also performed through the DAVID website (https://david.ncifcrf.gov). The results are listed in Fig. 6b, wherein 5 BPs for 183 genes were observed including antigen processing and presentation, defense response to virus, innate immune response, negative regulation of viral genome replication, and positive regulation of catalytic activity. As for KEGG analysis, the proteasome, Epstein-Barr virus infection, antigen processing and presentation, insulin signaling pathway, and phagosome were included. In summary, all the above results indicated that HLA-F could affect glioma-related immune activities.

## Discussion

Gliomas are the most common type of primary malignant tumors and possess high cancer-associated mortality rate [24]. According to the Stupp protocol, surgery and postoperative radiotherapy plus chemotherapy have improved the survival of patients with gliomas; however, the outcome of most of the glioma patients remained poor [25]. Slow progression in the extension of patients’ lives highlighted the urgent need for new and effective treatments for gliomas [26].

Immunotherapies for gliomas were considered to have a bright future. Anti-glioma immunotherapy is a general term that encompassed various strategies that are intended to stimulate the patient’s innate and adaptive immune system against gliomas and promote immune-mediated anti-glioma responses [27]. The TCGA network describes a robust gene expression-based molecular classification of gliomas into proneural, neural, classical, and mesenchymal subtypes. Gliomas in the mesenchymal group are more malignant than others [23]. In this study, we retrospectively analyzed 593 glioma patients from the CGGA RNA-seq set and GSE 16011 array set. Our findings indicated that HLA-F significantly predicted the OS in all grade gliomas and GBM. Moreover, the mRNA expression level remarkably varied in different grades and IDH1 status groups. Next, we found that HLA-F was upregulated in the mesenchymal group, enlightening HLA-F as a biomarker for the mesenchymal subtype, a more malignant phenotype and worse prognosis.

HLA complex, also known as the major histocompatibility complex (MHC), consists of three groups: HLA-I (e.g., HLA-A, HLA-E, and HLA-F), HLA-II (e.g., HLA-DR, HLA-DP, and HLA-DQ), and HLA-III [28]. Former studies indicated that HLA-F expression could protect the trophoblasts from NK- and CTL-mediated lysis in the maternal uterine environment [29]. Moreover, HLA-F, as an important immunosuppressive molecule, is exhibited differently in various types of tumors. In hepatocellular carcinoma, HLA-F expression was significantly correlated with the degree of lymphatic or venous invasion [30]. Furthermore, in esophageal squamous cell carcinoma, higher HLA-F expression indicated worse survival [31].

HLA-A, HLA-B, and HLA-C mainly present antigens to CD8 T cells and regulate various immunologic functions. HLA-E and HLA-F mainly regulate NK cell function through NK cell receptors. HLA-G is highly expressed on the surface of cytotrophoblasts and placenta and presented self-polypeptides. We found that HLA-F was correlated with most of the HLA complexes, such as HLA-A, HLA-B, HLA-C, and HLA-E. A variety of HLA molecules were enriched in gliomas to participate in the immunosuppressive state of CD8 and NK cells. Furthermore, HLA-F-positive gliomas were associated with immune response, indicating that the function of HLA-F in gliomas involved the regulation of immune status of tumor tissues.

Lack of HLA-F could render gliomas to be susceptible to elimination by NK cells. In our opinion, during the development of gliomas, glioma-specific and microenvironment cue-induced HLA-F expression could be modulated on glioma cells. Higher HLA-F expression in glioma cell surface provided immunoinhibitory effects and protected malignant glioma cells from elimination by NK cells, inducing the growth of glioma cells. HLA-F may act as a novel target of glioma immunotherapy.

## Conclusion

In conclusion, to the best of our knowledge, this was the first study to investigate the HLA-F expression levels in gliomas. The results demonstrated that HLA-F could be considered as a novel immunotherapy target in gliomas.

## Abbreviations

AUC: Area under the curve
BP: Biological process
CGGA: Chinese Glioma Genome Atlas
CNS: Central nervous system
GBM: Glioblastoma
GO: Gene Ontology
HLA: Human leukocyte antigen
IDH1: Isocitrate dehydrogenase 1
KEGG: Kyoto Encyclopedia of Genes and Genomes
NK cells: Natural killer cells
OS: Overall survival
WHO: World Health Organization
